# Temporal Associations Between Cognitive Impairment and Depression in Older Adults: A Longitudinal Analysis

**DOI:** 10.3390/ejihpe15070132

**Published:** 2025-07-12

**Authors:** Jesús Herrera-Imbroda, Vera Carbonell-Aranda, Gloria Guerrero-Pertiñez, Pilar Basnestein-Fonseca, Peter Anderberg, Esperanza Varela-Moreno, Antonio Cuesta-Vargas, Maite Garolera, Evi Lemmens, Johan Sanmartin Berglund, Fermin Mayoral-Cleries, Jessica Marian Goodman-Casanova, Jose Guzman-Parra

**Affiliations:** 1 Mental Health Department, The Biomedical Research Institute of Málaga and Nanomedicine Plattform (IBIMA Bionand Platform), University Regional Hospital of Málaga, 29009 Málaga, Spain; jesus.herrera.imbroda.sspa@juntadeandalucia.es (J.H.-I.); vera.carbonell.sspa@juntadeandalucia.es (V.C.-A.); esperanza.varela@ibima.eu (E.V.-M.); fermin.mayoral.sspa@juntadeandalucia.es (F.M.-C.); jmariangoodman@gmail.com (J.M.G.-C.); jose.guzman.parra.sspa@juntadeandalucia.es (J.G.-P.); 2 Cudeca Foundation, The Biomedical Research Institute of Málaga and Nanomedicine Plattform (IBIMA Bionand Platform), 29631 Málaga, Spain; pilarbarnestein@cudeca.org; 3 Department of Health, Blekinge Institute of Technology, 37179 Karlskrona, Sweden; peter.anderberg@bth.se (P.A.); johan.sanmartin.berglund@bth.se (J.S.B.); 4 Department of Physiotherapy, The Biomedical Research Institute of Málaga and Nanomedicine Plattform (IBIMA Bionand Platform), University of Malaga, 29071 Málaga, Spain; acuesta@uma.es; 5 Brain, Cognition and Behavoir-Clinical Research, Consorci Sanitari de Terrasa, 08227 Barcelona, Spain; mgarolera@cst.cat; 6 Faculty of Health, Centre of Expertise Health Innovation, University Colleges Leuven-Limburg (UCLL), 3600 Genk, Belgium; evi.lemmens@ucll.be

**Keywords:** aging, dementia, mild cognitive impairment, depression

## Abstract

Depression and cognitive impairment frequently co-occur in older adults, but their temporal relationship remains unclear. While depression is often considered a risk factor for cognitive decline, evidence is mixed, particularly in individuals with mild cognitive impairment or early dementia (MCI/ED). This study analyzed longitudinal data from 1086 participants (M = 74.49, SD = 7.24) in the SMART4MD clinical trial, conducted in Spain and Sweden over 18 months, with assessments every six months. Cognitive impairment was measured using the Mini-Mental State Examination, and depression was assessed with the Geriatric Depression Scale-15. Findings revealed a concurrent association between depressive symptoms and cognitive impairment. In regression mixed analysis, depression levels predicted increased cognitive decline over time, but no evidence was found for cognitive impairment predicting future depression. These associations were confirmed using a bivariate latent growth curve model with cross-lagged paths, which revealed early but attenuating bidirectional effects between depression and cognition. These results highlight depression as a medium-term risk factor for cognitive decline, emphasizing the importance of addressing depressive symptoms to mitigate cognitive deterioration in MCI/ED populations.

## 1. Introduction

Cognitive impairment is responsible for an important disability burden at the individual and social level (Pérez Palmer et al., 2022). Cognitive impairment frequently co-occurs with depression (Ismail et al., 2017). However, the causal and temporal relationship between cognitive impairment and depression is complex and not clearly understood (Hill et al., 2016; Jorm, 2000), leading to mixed findings (Bassuk et al., 1998; Baune et al., 2007; Chodosh et al., 2007; Ganguli et al., 2006; Geerlings et al., 2000; Paterniti et al., 2002; Sachs-Ericsson et al., 2005), probably due to different characteristics of the samples and methodology used in the studies. Numerous studies have shown the relationship between the conditions in older adults. However, there are few studies on this relationship in samples of older adults who exhibit cognitive decline or early stages of dementia but do not present clinically relevant depressive states. Studying the temporal relationship in this type of sample can help clarify the relationship between these two conditions once cognitive decline has started. There are various observations on the temporal relationship between cognitive decline and depression that would support different hypotheses: (1) Cognitive decline precedes depressive symptoms, which would support the hypothesis that depression is a reaction to cognitive decline. Some studies find that cognitive decline precedes depression and that there may be a depressive effect of cognitive decline (Brailean et al., 2017; Nys et al., 2006; Yu et al., 2018). (2) Depression precedes cognitive decline, indicating that depression would be a prodrome of cognitive decline. There is a large number of studies finding that depression precedes cognitive decline (Gallagher et al., 2016; Hofbauer & Rodriguez, 2023; W. Huang et al., 2022; Lara et al., 2017; Park et al., 2023; Paterniti et al., 2002; Vinkers et al., 2004). However, not all studies find this association (Ganguli et al., 2006). (3) Depression in the medium- or long-term influences the temporal evolution of cognitive decline, indicating that depression is a risk factor for the onset of cognitive decline. A meta-analysis has found that a history of depression throughout life is more related to dementia than late-onset depression (Ownby et al., 2006). Other studies have also determined that depression influences the evolution of cognitive decline over time in long-term follow-ups (Brenowitz et al., 2021; Panza et al., 2009). (4) Cognitive decline in the medium- and long-term influences the temporal evolution of depression, indicating that cognitive decline is a risk factor for the development of depression. Some studies have observed a relationship between cognitive decline and the medium- to long-term evolution of depression (Jajodia & Borders, 2011), but more studies have not found this relationship (Z. Wu et al., 2021). (5) The co-occurrence of both conditions could indicate that cognitive decline and depression share the same risk factors, or that depression lowers the threshold for the manifestation of cognitive decline, or that cognitive decline is a symptom or characteristic of depression. The concurrent relationship between depression and cognitive decline is a well-established fact throughout the literature and has been confirmed in numerous studies (Ismail et al., 2017; Rock et al., 2014).

Despite this studied association between cognitive decline and depression, the pathogenic explanations for it are still not entirely clear, which has been the subject of a recent systematic review (Botto et al., 2022). Thus, taking Alzheimer’s Disease (AD) as a model, the authors have found findings that suggest a bidirectional causal inference between the two phenomena. At first, during the onset of cognitive impairment, depression may arise as a psychological reaction to AD due to coping difficulties related to the loss of functional capacity experienced by these patients (Holtzer et al., 2005; Wilson et al., 2008; Zahodne et al., 2013). At the same time, neurodegeneration of the areas and circuits that manage emotions can lead to anxiety and depression in AD. For example, some biomarkers that have been associated with depression in AD are a decrease in Aβ42 and an increase in t-tau and p-tau in CSF (Banning et al., 2021). Finally, in the later stages of AD, cognitive impairment reduces emotional responses and their expression, as evidenced by the fact that affective symptoms tend to reduce as the disease progresses (Milwain & Nagy, 2005; Y. Wu et al., 2020). Thus, the development of AD seems to stop the continuity of the depressive state due to the deterioration of memory and executive control (Wilson et al., 2010). It seems clear that an in-depth analysis of this relationship would provide policymakers and health care providers with guidance for the prevention and management of these patients.

Thus, the aim of the study is to verify the bidirectional temporal relationship between depression and cognitive decline among older adults with mild cognitive impairment and/or early stages of dementia who do not present a depressive episode nor moderate or severe depressive symptoms.

## 2. Materials and Methods

### 2.1. Design

Longitudinal study based on a secondary analysis from the SMART4MD randomized multicenter clinical trial (ClinicalTrials.gov identifier NCT03325699). The aim of the study was to create digital software (Support Monitoring and Reminder for Mild Dementia, SMART4MD) for a tablet and to test if it influenced the quality of life of participants and their caregivers. The intervention group used the SMART4MD tablet-based app, which offered functionalities for cognitive stimulation, medication reminders, calendar appointments, and general health information. The control group received care as usual, without digital support. Results from the original trial showed that although the intervention did not significantly improve overall quality of life compared to control, it was positively evaluated by users and associated with improved adherence to medication and reduced caregiver burden in some subgroups. Detailed information about the trial is available in the protocol (Anderberg et al., 2019). For the trial, there were four assessments every 6 months over an 18-month period where depression and cognitive impairment were measured.

### 2.2. Setting

The trial was conducted in two countries: Spain and Sweden, and three centers: Consorci Sanitari de Terrassa (Catalonia, Spain), Servicio Andaluz de Salud (Andalusia, Spain), and the Blekinge Institute of Technology (Karlskrona, Sweden).

### 2.3. Participants

In total, 1086 participants were included in the study. The number of participants that completed the different assessments and the sociodemographic characteristics of the participants are shown in Table 1. The sample was selected using a non-probabilistic consecutive sampling method from primary care and memory clinics in three centers in Spain and Sweden as outlined in the SMART4MD trial protocol (Anderberg et al., 2019). The inclusion criteria were as follows: (a) a score of 20 to 28 points on the Mini Mental State Examination (MMSE), (b) an experience of memory problems over a substantial period of time (more than 6 months), (c) age > 55 years, (d) to be home care recipients, (e) to have an informal caregiver, (f) to take prescribed medication and to be in charge of it, and (g) not to have conditions that reduce their physical ability to use a touchscreen App. The exclusion criteria were as follows: (a) to have a terminal illness with less than three years of expected survival, (b) to score above 11 on the Geriatric Depression Scale (GDS-15) or (c) to have another known significant cause of disease as an explanation for cognitive impairment such as substance abuse, bipolar disorder, schizophrenia, or developmental disorders. Data were collected during four face-to-face interviews over an 18-month period (baseline, 6, 12, and 18 months). Trained research staff, blinded to group allocation, conducted structured assessments using linguistically validated Spanish and Swedish versions of the MMSE and GDS-15. Responses were recorded electronically and underwent consistency checks by both site-level coordinators and the central study team. A detailed description of the participants is in Table 1.

### 2.4. Measures

Cognitive function was assessed with the MMSE (Folstein et al., 1975) to estimate the severity of cognitive impairment. Depression was measured with the GDS-15 (Yesavage et al., 1982). It is a widely used scale to assess geriatric depression with 15 items and a range score of 0–15. Also, sociodemographic data were taken into account, namely age, gender and educational level.

### 2.5. Statistical Analysis

Descriptive statistics were used to describe the sample. Mixed regression models were used to analyze the longitudinal nature of the data, being the individual random effect. Concurrent and lagged models were used to analyze the relationship between depressive symptoms and cognitive impairment. Two models were used: (1) in which the dependent variable was cognitive impairment, and the independent variable was depression, and (2) another, in which the dependent variable was depression and the independent variable was cognitive impairment. Regarding independent variables, two variables were introduced: (1) the mean of the individual throughout the evaluation period (time-invariant variable) and (2) a measure for each evaluation carried out (time-varying variable) which was centered to that person’s mean. In the lagged model, the previous measurement of the independent variable (t − 1) was used as a predictor of the value of the dependent variable in the next assessment. Models with random intercepts, random slopes and autoregressive correlation were tested and selected the models with less error, or the most parsimonious, if there were no significant differences between the models. In the regression models, age, gender, time of assessment, educational level and group in the clinical trial were included as confounding variables. Similarly, the interactions between the independent variable (time-invariant and time-variant) and time were included in the models. To quantify the explanatory contribution of the interactions, we calculated the marginal R^2^ (variance explained by fixed effects) following the method proposed by (Nakagawa & Schielzeth, 2013). These analyses were conducted using the nlme package in R.

To examine the longitudinal relationship between depressive symptoms and cognitive performance, we specified a bivariate latent growth curve model with freely estimated slopes and cross-lagged paths across four time points (baseline, 6, 12, and 18 months). The model included two latent trajectories (intercept and slope) for both depression and cognition. In addition to modeling the overall growth trajectories, we incorporated lagged effects to evaluate the directional influence of one domain on the other over time (e.g., depression at baseline predicting cognition at 6 months, and vice versa). Covariates (age, gender, education, and intervention group) were included as predictors of the latent factors. The model was estimated using robust maximum likelihood (MLR) with full information maximum likelihood (FIML) for handling missing data. Model fit was evaluated using standard indices: Comparative Fit Index (CFI), Tucker–Lewis Index (TLI), Root Mean Square Error of Approximation (RMSEA), Standardized Root Mean Square Residual (SRMR), and the chi-square test. The analyses were conducted using the lavaan package in R.

The level of significance was set at 0.05. For statistical analysis, the R-Studio 2023.03.0 + 386 program with version R 4.3.0 was used.

## 3. Results

In the concurrent multivariate model, with cognitive decline as the dependent variable, statistically significant associations were found with average depression during the different assessments (B = −0.136, *p* < 0.001), and with the interaction between time and average depression (B = −0.038, *p* = 0.011) and time and time-varying depression (B = −0.072, *p* = 0.004). More information on the model appears in Table 2.

When depression was used as the dependent variable, the average cognitive decline (B = −0.132, *p* < 0.001) was associated with this variable, but there were no significant differences in the interaction between average cognitive decline and time (B = 0.001, *p* = 0.931) nd time-varying cognitive decline (B = −0.032, *p* = 0.128). More information appears in Table 3.

Regarding the lagged models, there was no association between the previous depression value (B = −0.024, *p* = 0.632) and the cognitive decline of the next assessment, nor vice versa (B = −0.033, *p* = 0.457). The interaction between average depression and time remained nearly significant (B = −0.040, *p* = 0.058), as well as between time-varying depression and time (B = 0.089, *p* = 0.072). The interaction was significant between time-varying cognitive decline and time (B = 0.077, *p* = 0.044), but not between average cognitive decline and time (B = −0.002; *p* = 0.902). More information about the lagged models appears in Table 4 and Table 5.

**Figure 1:**
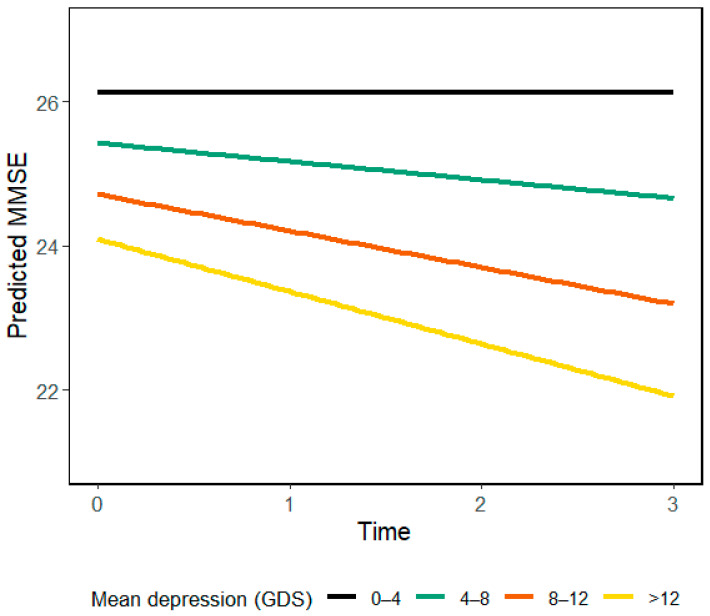
Predicted MMSE trajectories over time by average level of depressive symptoms (GDS). Individuals with higher average depression scores showed a more pronounced cognitive decline, with the steepest trajectory observed in those scoring above 12 on the GDS. In contrast, individuals with low or no depressive symptoms (GDS 0–4) maintained higher and more stable cognitive performance.

The inclusion of the depression × time interaction term led to an increase in the marginal R^2^ from 0.031 to 0.041. This suggests that the interaction accounts for approximately 1% of the variance in cognitive functioning over time, The information of the results of the mixed models without interaction is shown in the Supplementary material (Supplementary Tables S1–S4). As illustrated in Figure 1, individuals with higher average levels of depression showed a faster decline in MMSE scores over time. Specifically, those in the highest interval (>12 on the GDS) showed a markedly steeper trajectory compared to those with low or no symptoms (GDS 0–4). This suggests that persistent depressive symptoms may contribute to accelerated cognitive decline.

The bivariate latent growth curve model showed excellent fit to the data (Shown in Table 6). A significant negative association was observed between baseline levels of depressive symptoms and cognitive performance (β = −0.127, *p* = 0.002), indicating that participants with greater baseline cognitive impairment also exhibited more depressive symptoms at that time. In addition, baseline depression levels significantly predicted later cognitive decline (β = −0.146, *p* = 0.043), suggesting that participants with more depressive symptoms at baseline tended to experience greater deterioration in cognitive performance over time. However, no significant association was found between the rate of change in depression and the rate of change in cognition (r = −0.641, *p* = 0.221), nor between baseline cognitive functioning and subsequent change in depressive symptoms (r = −0.438, *p* = 0.142). The model included cross-lagged effects to examine temporal influences between cognition and depression. Early bidirectional effects were observed. Baseline depression negatively predicted cognitive performance at six months (β = 0.059, *p* = 0.007), and this pattern remained marginally significant at 12 months (*p* = 0.057), although it attenuated by 18 months. Conversely, baseline cognitive performance also predicted higher levels of depressive symptoms at six months (β = 0.035, *p* = 0.016), with a marginal trend at 12 months (*p* = 0.058), but no significant effect at 18 months (*p* = 0.327). Further details on the model can be found in Table 6.

## 4. Discussion

The study found a significant concurrent association between symptoms of depression and cognitive decline. Regarding the temporal relationship between symptoms of depression and cognitive decline in mixed regression analysis, the measure of each six months prior did not predict the subsequent measure, respectively. There was a relationship between depression over the study period and an increase in the time of cognitive decline. In contrast, the evidence supporting an effect in the opposite direction was weaker and inconsistent. Although early bidirectional effects were observed in the latent growth curve model (e.g., baseline cognition predicting depression at 6 months), these effects attenuated over time and were not statistically significant at later assessments. These data would support the hypothesis that depression is a medium-term risk factor for cognitive decline, but provide limited support for the reverse hypothesis that cognitive decline contributes meaningfully to the development or progression of depressive symptoms in individuals already experiencing cognitive deficits.

In line with our results, several studies have found that depression is a risk factor for subsequent cognitive decline and dementia (Cooper et al., 2015; Da Silva et al., 2013; Gallagher et al., 2016; John et al., 2019; Yuan et al., 2023), although some studies find contrary data (Becker et al., 2009; Brailean et al., 2017; Bunce et al., 2012; Freire et al., 2017). On the other hand, other studies, including a meta-analysis, have not found evidence that cognitive decline is a risk factor for depression (C. Q. Huang et al., 2011), although there is also evidence to the contrary (Mirza et al., 2017).

The mixed results found in the literature may be related to the complexity of the relationship found in this study. Although depressive symptoms and cognitive performance were strongly associated at each time point, prior depression scores did not consistently predict subsequent cognitive decline. This may be due to the fluctuating nature of depressive symptoms and their strong concurrent relationship with cognition, which could obscure their predictive value in short time intervals. However, average depression levels across all assessments—reflecting more stable and persistent symptoms— and baseline scores were significantly associated with accelerated cognitive decline over time. This suggests that sustained depressive symptoms, rather than transient episodes, may play a more relevant role in cognitive deterioration.

Among the limitations of the study is that it is a secondary study of a clinical trial, and therefore, there may be biases in the selection of participants that do not represent the general population. Also, the follow-up time was relatively short, only 18 months, so conclusions cannot be drawn about long-term outcomes. Likewise, only four evaluations were conducted, and there may be biases due to variability in depression over time. Thus, the short follow-up period and the limited number of assessment points likely affect their ability to detect true cognitive decline over time. The Mini-Mental State Examination for assessing cognitive decline is a test that has some limitations, as it sometimes underestimates cognitive decline in certain populations and overestimates it in others.

An important limitation of this study is the lack of key indicators of health and functional status, such as Activities of Daily Living (ADL), physical comorbidities, or physical activity levels. These variables may play a mediating or moderating role in the relationship between depression and cognitive decline, and their absence may reduce the accuracy of estimates or bias the effect size. Future studies are encouraged to integrate functional assessments and biomarkers to clarify the mechanisms linking depression and cognitive deterioration.

## 5. Conclusions

This study underscores the importance of identifying and promptly treating depressive symptoms in individuals with mild cognitive impairment or early stages of dementia, as these symptoms are related to the progression of cognitive decline over time. Moreover, the study indicates that in a population with low levels of depression, like the one in this study, depressive symptoms were linked to increased cognitive decline. This suggests that clinicians should be attentive to subsyndromal depressive symptoms, which are often overlooked, to prevent further cognitive deterioration in at-risk populations already experiencing cognitive decline. However, further studies are needed on the complex relationship between cognitive decline and depression to help explain the causal relationship between these two phenomena.

## Figures and Tables

**Table 1:** Characteristics of the sample.

Characteristics	Baseline n = 1083	6 Months n = 848	12 Months n = 753	18 Months n = 650
Age, M (SD)	74.49 (7.24)	74.04 (7.17)	75.05 (7.04)	73.95 (6.845)
Gender (Women), n (%)	576 (53.2)	436 (51.4)	382 (50.7)	320 (49.2)
Educational Level, n (%)				
Elementary school	646 (59.9)	507 (60.1)	444 (59.3)	380 (58.6)
Secondary school	225 (20.9)	168 (19.9)	151 (20.2)	132 (20.4)
Higher education	207 (19.2)	169 (20.0)	154 (20.6)	136 (21.0)
Geriatric Depression Scale, M (SD)	3.01 (2.81)	2.92 (2.99)	2.84 (2.95)	2.77 (3.03)
Mini Mental State Examination, M (SD)	25.45 (2.46)	25.50 (3.71)	25.48 (4.03)	25.80 (4.07)

**Table 2:** Concurrent Mixed Regression model using cognitive impairment (MMSE) as the dependent variable.

Models	Concurrent Model				
Fixed Effects	B	SE	Df	t	*p*
Depression (time-varying)	−0.005	0.037	2082	−0.148	0.881
Average Depression (time-invarying)	−0.136	0.029	1076	−4.777	<0.001
Time	−0.083	0.146	1076	−0.566	0.572
Intervention Group (control reference)	−0.079	0.147	1076	−0.538	0.590
Age	−0.073	0.01	2082	−7.090	<0.001
Gender (female reference)	−0.310	0.149	1076	−2.086	0.037
Educational Level					
Elementary school (reference)					
Secondary school	−0.015	0.097	2082	−0.153	0.878
Higher education	0.123	0.101	2082	1.208	0.227
Average Depression × Time	−0.038	0.015	2082	−2.535	0.011
Depression (time-varying) × Time	−0.072	0.025	2082	−2.895	0.004

Note: AIC (14,486.17), BIC (14,577.03), logLik (−7228.083).

**Table 3:** Concurrent Mixed Regression model using depression (GDS) as the dependent variable.

Models	Concurrent Model				
Fixed Effects	B	SE	Df	t	*p*
* MMSE (time-varying)	−0.0409	0.034	2082	−1.207	0.227
MMSE average (time-invarying)	−0.132	0.027	1076	−4.883	<0.001
Time	0.041	0.277	2082	0.148	0.882
Intervention Group (control reference)	−0.150	0.157	1076	−0.953	0.341
Age	−0.067	0.011	2082	−6.157	<0.001
Gender (female reference)	0.68	0.158	1076	4.293	<0.001
Educational Level					
Elementary school (reference)	0.679	0.158	1076	4.293	<0.001
Secondary school	−0.079	0.095	2082	−0.831	0.406
Higher education	−0.193	0.098	2082	−1.207	0.228
MMSE average × Time	−0.001	0.011	2082	−0.085	0.931
MMSE (time-varying) × Time	−0.032	0.021	2082	−1.524	0.128

* MMSE: Mini Mental State Examination. Note: AIC (14,022.17), BIC (14,113.04), logLik (−6996.087).

**Table 4:** Lagged Mixed Regression model using cognitive impairment (MMSS) as the dependent variable.

Models	Lagged Model				
Fixed Effects	B	SE	Df	t	*p*
Depression (time-varying)	−0.024	0.050	1249	−0.479	0.632
Average Depression (time-invarying)	−0.204	0.047	857	−4.302	0.001
Time	−0.018	0.076	1249	0.239	<0.001
Intervention Group (control reference)	−0.013	0.243	857	−0.052	0.958
Age	−0.096	0.017	1249	−5.611	<0.001
Gender (Woman reference)	−0.770	0.246	857	−3.133	0.002
Educational Level					
Elementary school (reference)					
Secondary school	0.090	0.134	1249	0.672	0.502
Higher education	0.158	0.139	1249	1.138	0.255
Average Depression × Time	−0.040	0.021	1249	−1.897	0.058
Depression (time-varying) × Time	0.089	0.049	1249	1.800	0.072

Note: AIC (10,301.51), BIC (10,386.3), logLik (−5135.754).

**Table 5:** Lagged Mixed Regression model using depression (GDS) as the dependent variable.

Models	Concurrent Model				
Fixed Effects	B	SE	Df	t	*p*
* MMSE (time-varying)	−0.033	0.045	1177	−0.743	0.457
MMSE average (time-invarying)	−0.164	0.035	858	−4.746	<0.001
Time	0.099	0.451	1177	0.219	0.826
Intervention Group (control reference)	−0.015	0.184	858	−0.083	0.934
Age	−0.074	0.013	1177	−5.714	<0.001
Gender (female reference)	0.638	0.185	858	3.438	0.006
Educational Level					
Elementary school (reference)					
Secondary school	−0.070	0.118	1177	−0.589	0.556
Higher education	−0.110	0.122	1177	−0.904	0.366
MMSE average × Time	−0.002	0.017	1177	−0.123	0.902
MMSE (time-varying) × Time	0.077	0.038	1177	2.019	0.044

* MMSE: Mini Mental State Examination. Note: AIC (9214.442), BIC (9287.479), logLik (−4594.221).

**Table 6:** Standardized estimates of the relationship between cognition and depression based on a latent growth curve model with cross-lagged effects (LGCM).

Type of Association	Standardized Estimate (β)	*p*-Value
Concurrent Associations		
Baseline associations (Intercept of Cognition/Intercept of Depression)	−0.127	0.002
Longitudinal associations (Slope of Cognition/Slope of Depression)	−0.641	0.221
Baseline cognition interaction with time (Intercept of Cognition/Slope of Depression)	−0.438	0.142
Baseline depression interaction with time (Intercept of Depression/Slope of Cognition)	−0.146	0.043
Cross-Lagged (Temporal) Associations		
Depression Baseline → Cognition at 6 months	0.059	0.007
Depression at 6 months → Cognition at 12 months	0.052	0.057
Depression at 12 months → Cognition at 18 months	0.055	0.084
Cognition Baseline → Depression at 6 months	0.035	0.016
Cognition at 6 months → Depression at 12 months	0.112	0.058
Cognition at 12 months → Depression at 18 months	0.13	0.327
Model Fit Indices		
Comparative Fit Index (CFI)	0.994	
Tucker–Lewis Index (TLI)	0.987	
RMSEA (90% CI: 0.017–0.041)	0.029	
Standardized Root Mean Square Residual (SRMR)	0.014	
χ^2^ (df = 26), *p*-value	49.97	0.003
Akaike Information Criterion (AIC)	28,975.08	
Bayesian Information Criterion (BIC)	29,224.22	

## Data Availability

The data are available upon reasonable request to the corresponding author.

## Supplementary Materials

The following supporting information can be downloaded at: https://www.mdpi.com/article/10.3390/ejihpe15070132/s1, Table S1: Concurrent Mixed Regression model using cognitive impairment (MMSE) as dependent variable (without the interactions); Table S2: Concurrent Mixed Regression model using depression (GDS) as dependent variable (without the interactions); Table S3: Lagged Mixed Regression model using cognitive impairment (MMSE) as dependent variable (without the interactions); Table S4: Lagged Mixed Regression model using depression (GDS) as dependent variable (without the interactions).

## Abbreviations

The following abbreviations are used in this manuscript:

MCI/ED	Mild cognitive impairment or early dementia
SMART4MD	Support Monitoring and Reminder for Mild Dementia
MMSE	Mini Mental State Examination
GDS-15	Geriatric Depression Scale (GDS-15)
