# Supplementary material for: Temporal Associations Between Cognitive Impairment and Depression in Older Adults: A Longitudinal Analysis
Source: Eur J Investig Health Psychol Educ. 2025 Jul 12;15(7):132. doi: 10.3390/ejihpe15070132 (PMC12293492; doi:10.3390/ejihpe15070132)
Supplement: Supplementary file 1 [file ejihpe-15-00132-s001.zip › ejihpe-3604260-supplementary.pdf]

Concurrent and Lagged models without interactions.

Supplementary Table S1. Concurrent Mixed Regression model using cognitive impairment (MMSE) as dependent variable (without the interactions)

Model	Concurrent model				
**Fixed Effects**	B	SE	Df	t	p
Depression (time varying)	-0.097	0.023	2084	-4.305	<0.001
Average Depression(time invarying)	-0.121	0.028	1076	-4.354	<0.001
Time	-0.168	0.040	2084	-4.198	<0.001
Intervention Group (Control reference)	-0.077	0.146	1076	-0.527	0.599
Age	-0.073	0.010	2084	-7.127	<0.001
Gender (Woman reference)	-0.300	0.149	1076	-2.016	0.044
Educational Level					
Secondary school	-0.011	0.097	2084	-0.113	0.910
Higher education	0.123	0.102	2084	1.213	0.225

Note: AIC (14484.51), BIC (14563.27), logLik (-7229.256)

Supplementary Table S2. Concurrent Mixed Regression model using depression (GDS) as dependent variable (without the interactions)

Model	Concurrent model				
**Fixed Effects**	B	SE	Df	t	p
MMSE(time varying)	-0.081	0.019	2084	-4.386	<0.001
MMSE average(time invarying)	-0.132	0.026	1076	-5.144	<0.001
Time	0.018	0.029	2084	0.629	0.530

Intervention Group (Control reference)	-0.150	0.157	1076	-0.957	0.339
Age	-0.068	0.010	2084	-6.179	<0.001
Gender (Woman reference)	0.680	0.158	1076	4.294	<0.001
Educational Level					
Elementary school (reference)					
Secondary school	-0.088	0.094	2084	-0.935	0.350
Higher education	-0.197	0.098	2084	-2.014	0.044

Note: AIC (14007.34), BIC (14086.0), logLik (-6990.668)

Supplementary Table S3. Lagged Mixed Regression model using cognitive impairment (MMSE) as dependent variable (without the interactions)

Model	**Lagged model**				
**Fixed Effects**	B	SE	Df	t	p
Depression (time varying)	0.042	0.031	1251	1.327	0.184
Average Depression (time invarying)	-0.204	0.047	857	-4.325	<0.001
Time	-0.123	0.052	1251	-2.364	0.018
Intervention Group (Control reference)	-0.012	0.243	857	-0.051	0.959
Age	-0.095	0.017	1251	-5.564	<0.001
Gender (Woman reference)	-0.773	0.246	857	-3.146	0.002
Educational Level					
Elementary school (reference)					
Secondary school	0.070	0.134	1251	0.522	0.602
Higher education	0.139	0.138	1251	1.003	0.316

Note: AIC (10294.63), BIC (10368.13), logLik (-5134.315)

Supplementary Table S4. Lagged Mixed Regression model using depression (GDS) as dependent variable (without the interactions).

Model			Concurrent model		
**Fixed Effects**	**B**	**SE**	**Df**	**t**	**p**
MMSE (time varying)	0.028	0.023	1179	1.184	0.236
MMSE average (time invarying)	-0.152	0.029	858	-5.159	<0.001
Time	0.043	0.044	1179	0.978	0.328
Intervention Group (Control reference)	-0.015	0.184	858	-0.096	0.923
Age	-0.074	0.013	1179	-5.727	<0.001
Gender (Woman reference)	0.638	0.185	858	3.432	0.006
Educational Level					
Elementary school (reference)					
Secondary school	-0.055	0.118	1179	-0.472	0.636
Higher education	-0.100	0.122	1179	-0.820	0.412

* MMSE: Mini Mental State Examination. Note: AIC (9204.098), BIC (9265.909), logLik (-4591.049)
